# LXR agonist improves peripheral neuropathy and modifies PNS immune cells in aged mice

**DOI:** 10.1186/s12974-022-02423-z

**Published:** 2022-02-26

**Authors:** Chaitanya K. Gavini, Nadia Elshareif, Gregory Aubert, Anand V. Germanwala, Nigel A. Calcutt, Virginie Mansuy-Aubert

**Affiliations:** 1 Cell and Molecular Physiology, Stritch School of Medicine, Loyola University Chicago, Maywood, IL 60153 USA; 2 Department of Neurological Surgery, Loyola University Medical Center, Maywood, IL 60153 USA; 3 Departement of Internal Medicine, Division of Cardiology, Loyola University Medical Center, Maywood, IL 60153 USA; 4 Department of Pathology, University of California San Diego, La Jolla, CA 92093 USA

**Keywords:** Liver X receptors, GW3965, Peripheral neuropathy, Aging

## Abstract

**Background:**

Peripheral neuropathy is a common and progressive disorder in the elderly that interferes with daily activities. It is of importance to find efficient treatments to treat or delay this age-related neurodegeneration. Silencing macrophages by reducing foamy macrophages showed significant improvement of age-related degenerative changes in peripheral nerves of aged mice. We previously demonstrated that activation of the cholesterol sensor Liver X receptor (LXR) with the potent agonist, GW3965, alleviates pain in a diet-induced obesity model. We sought to test whether LXR activation may improve neuropathy in aged mice.

**Methods:**

21-month-old mice were treated with GW3965 (25 mg/Kg body weight) for 3 months while testing for mechanical allodynia and thermal hyperalgesia. At termination, flow cytometry was used to profile dorsal root ganglia and sciatic nerve cells. Immune cells were sorted and analyzed for cholesterol and gene expression. Nerve fibers of the skin from the paws were analyzed. Some human sural nerves were also evaluated. Comparisons were made using either *t* test or one-way ANOVA.

**Results:**

Treatment with GW3965 prevented the development of mechanical hypersensitivity and thermal hyperalgesia over time in aged mice. We also observed change in polarization and cholesterol content of sciatic nerve macrophages accompanied by a significant increase in nerve fibers of the skin.

**Conclusions:**

These results suggest that activation of the LXR may delay the PNS aging by modifying nerve-immune cell lipid content. Our study provides new potential targets to treat or delay neuropathy during aging.

**Supplementary Information:**

The online version contains supplementary material available at 10.1186/s12974-022-02423-z.

## Background

Aging-related peripheral neuropathy and neuropathic pain contributes significantly to decreased quality of life in the elderly. Surveys report a prevalence of neuropathy and neuropathic pain of between 20 and 58% in people aged 60 and above, with prevalence directly proportional to age [1–4]. With age, the peripheral nervous system (PNS) undergoes pathophysiological changes that are also observed in people suffering with neuropathy, such as atrophy of large, myelinated fibers and thinning of the myelin sheath [5–7]. Some patients aged or otherwise develop peripheral neuropathies without a clear diagnosis, and the sural nerve biopsy can provide a diagnosis for the neuropathy [8, 9]. Studies from others and ours propose that neuropathy is significantly associated with lipid homeostasis in the PNS [6, 8, 10–12]. Understanding lipid metabolism in cells of the PNS is critical for understanding the pathophysiology of and developing novel targeted treatments for neuropathy.

Many forms of disease-initiated peripheral neuropathy share a common pathogenic mechanism that involves modifications to neuro-immune interactions mediated by inflammatory signals [13]. Immune cells expressing CD45 and CD11B enter the PNS from circulation and help to maintain the integrity of the nerve, in diseases models, they were shown to play a role in both regeneration and degeneration [14]. Damage to the nerve changes resident immune system that may be quantitative—loss of CD45 + leukocytes or increased infiltration of CD45 + leukocytes during inflammation-, or qualitative—change of macrophage phenotype [15]. Reduced recruitment of macrophages to the PNS, or their impaired capacity to phagocytosis results in impaired regeneration and maintenance of nerve integrity [16]. MHC class II (IA/IE) expressing inflammatory macrophages (as referred as M1), are less capable of phagocytosis than M2 macrophages (expressing CD206). Neuronal inflammation is implicated in the pathogenesis of several neurodegenerative diseases [17]. Recent studies have demonstrated that an increase in the number of macrophages in the dorsal root ganglia (DRG) and the sciatic nerve (SN) alters pain perception [18–20]. Macrophages and neutrophils are pivotal in clearance of debris during Wallerian degeneration following physical nerve injury [21, 22]; macrophages also form a bridge to facilitate migration endothelial cells and Schwann cells during nerve regrowth [23, 24]. Dysregulation of macrophage function can be detrimental to peripheral nerve. For example in Guillain–Barre syndrome, macrophages penetrate myelinating fibers at the nodes of Ranvier and promote axonal damage [25], while endoneurial macrophages are activated by Schwann cells and damage nerve fibers in a model of charcot-marie-tooth [26]. Targeting macrophages as an approach to treating age-related neuropathy is suggested by a report that macrophage silencing, achieved by blocking a cytokine receptor, reduced endoneurial foamy macrophages and significantly improved age-related degenerative changes in peripheral nerves of 24-month-old mice [6].

Circulating cholesterol and cholesterol pathways are linked to development and progression of neuropathy and studies have identified lipid-filled macrophages in the nerves of 24-month-old mice [12, 27, 28], which may reflect clearance of membrane debris. The clearance of such macrophages has been extensively studied in atherosclerosis, where dysfunction of lipid efflux from macrophages leads to inflammatory pathology [29]. Liver X receptors (LXR) are ligand-activated nuclear receptors that bind metabolites of cholesterol [30, 31], regulate cellular lipid efflux [32, 33] and have been involved in type II diabetic neuropathy [34]. In atherosclerosis, oxysterols derived from phagocytosis of apoptotic or necrotic cells activate the LXR pathway in naive macrophages, upregulating genes involved in cholesterol efflux, such as ATP binding cassette A1 (ABCA1) [35]. Overexpression of ABCA1 induces expression of the anti-inflammatory cytokines and other markers of M2 macrophages [36–38]. The role of LXR in immune cells located in the peripheral nerve system had not been investigated. In this study, we, therefore, treated aged mice with the selective LXR agonist GW3965 and evaluated some immune cell changes within the DRG and SN. We observed nerve regeneration and amelioration of neuropathic pain in aged mice treated with GW3965. Further experiments suggest that LXR activation changes nerve macrophage cholesterol efflux modifying the macrophage phenotypes.

## Methods

### Mice

All studies were conducted in accordance with recommendations in the Guide for the Care and Use of Laboratory Animals of the National Institutes of Health and the approval of the Loyola University Chicago Institutional Animal Care and Use Committee. C57BL/6 J mice (#000664, IMSR_JAX:000664) were obtained from Jackson laboratory (Maine, USA). All mice were housed under a 12:12 h light/dark cycle with free access to food (Teklad LM-485: Envigo, Indiana, USA) and water. All studies were performed using male mice with the experimenter blinded to treatment and recorded as per the ARRIVE guidelines [39].

### Human subjects

Informed consent was obtained from all human subjects (37yrs and 84yrs old, both females, patients undergoing leg amputations for trauma) prior to sural nerve sample collection in strict accordance with the rules and guidelines stipulated by the Loyola University Chicago Internal Review Board (IRB). All experimental protocols were approved by the Loyola University Chicago IRB (protocol # 210567020519). If a subject does not wish to participate, s/he is excluded from the study. During the surgery, the neurosurgeon resected the sural nerve (roughly 15 mm in length) for the study and the whole samples were processed for immune cells in the sciatic nerve as detailed below.

### In vivo agonist treatment

21-month-old mice (n = 10–12) were treated with vehicle or LXR agonist (GW3965; 25 mg/kg body weight) (Axon Medchem, Virginia, USA) by intraperitoneal injection twice weekly for 12 weeks (each i.p separated by 3 days), starting at 21 months of age. Tissues were rapidly dissected and processed or frozen in liquid nitrogen before analysis.

### Mechanical sensitivity

6 month (*n* = 6), 12 month (*n* = 6), vehicle or GW3965 treated 21–24 month-old mice (*n* = 10–12/group) were evaluated in a quiet room, at a constant temperature of 24 °C and acclimated to the von Frey chambers for at least 40 min, but not restrained in the chamber any longer than necessary to minimize stress and discomfort-induced behavioral variations. Mice were investigated for mechanical allodynia using phasic stimulation of the hind paws with von Frey filaments as described before [10]. Briefly, after acclimation to the testing chambers, mice were then subjected to iterative stimulation with calibrated von Frey filaments (0.16; 0.4; 1; 2; 4; 6; 8 g: North Coast Medical, California, USA). Filaments were applied 6 times for 1 at 1 s intervals with a 5 min break between each series of stimulations to the plantar surface of the mice hind limbs. A response to stimulation was defined as paw withdrawal or licking of the stimulated paw [40]. Response frequency for each filament was recorded and 50% threshold calculated. The test was performed once every 3 weeks for the vehicle or GW3965 treated age-matched mice for 12 weeks starting at 21 months to 24 months. A single trained investigator made all baseline and experimental measurements for these series of experiments while remaining blinded to the treatment groups.

### Thermal nociception

6 month (*n* = 6), 12 month (*n* = 6), vehicle or GW3965 treated 21–24 month-old mice (*n* = 6/group) were investigated for heat nociception using Plantar Test Apparatus (Hargreaves Method: IITC Life Science, California, USA) (SCR_012152) [40]. Briefly, after acclimation to testing chambers, tests were performed on the hind paw plantar surface using a focused, radiant heat light source with a built-in timer displaying reaction time in seconds. The heating rate was 1^ °^C/s and a cutoff time of 20 s was set to avoid tissue damage. The test was performed once every 3 weeks for the vehicle or GW3965 treated age-matched mice for 12 weeks starting at 21–24 months.

### Nerve fiber densities

Analysis of fiber density was performed routinely as described in detail elsewhere [40]. Briefly, footpads were collected from hind paws (*n* = 4–5/group) and transferred to Zamboni’s fixative for 4 h on ice before washing with PBS and embedding in paraffin block. 6 µm sections were cut using a rotary microtome (Leica, Illinois, USA) and mounted onto glass slides. Samples were immunostained with anti-PGP9.5 antibody (1:1000, #7863–0504, AB_2210505; AbD Serotec, California, USA) and nerve fiber density was quantified under blinded conditions by light microscopy at 40 × magnification. All nerve fragments in the epidermis and papillary dermis were counted for detection of nerve fiber terminal loss. The length of the paw skin was traced under light microscope and nerve density was reported as profiles/mm.

### Cell dissociation

Following euthanasia by isoflurane and cervical dislocation (*n* = 6–9/group), DRG and the maximal length of the SN (region identical between each mice and using the step-by-step approaches published elsewhere [41]) were dissected and placed in trypsin/collagenase A (1.25 mg/ml each) (#T1426, #C9407, respectively, Sigma-Aldrich, Missouri, USA) in PBS and incubated for 30 min at 37 °C in a 5% CO_2_ incubator. Digested tissues were dissociated into single cell suspension using fire polished glass pipettes before centrifuging at 300 *g* for 5 min. The resuspended cells were processed as described below.

### Flow cytometry

Cells dissociated from the DRG and whole SN were stained with fluorescently conjugated antibodies (CD45, CD11B, I-A/I-E, F4/80, CD206) (#157604, AB_2876536; #101212, AB_312795; #107650, AB_2566438; #123114, AB_893478; #141734, AB_2629637, respectively; BioLegend, California, USA) as previously described [42–44]. Briefly, dissociated single cells from DRG and SN were incubated in blocking solution (5% horse serum in PBS) for 30 min on ice. After pelleting the cells at 300 g for 5 min, above-mentioned fluorescently labelled antibodies were assayed at 1:1000 dilution and incubated for 1 h on ice. Cells were then washed and resuspended in blocking buffer before analyzing. Cells that were positive for both CD45 and CD11B were sorted from other dissociated cells for further analysis. Flow cytometry data were acquired using a BD FACS Aria III (BD Biosciences, California, USA) and data analyzed using FlowJo (BD Biosciences, California, USA).

### Quantitative PCR

mRNA was extracted from sorted CD45 + /CD11B + cells (*n* = 6–8/group) using Arcturus PicoPure RNA Isolation Kit (ThermoFisher, Massachusetts, USA) before generating cDNA using High Capacity cDNA Reverse Transcription Kit (ThermoFisher, Massachusetts, USA). For all genes of interest, qPCR was performed using Sybr green-based assay (Roche, Indiana, USA) using IDT primers (IDT technologies, Iowa, USA). 18 s was used to normalize data and quantification was done using ΔΔCT method with the mean value of the vehicle treated group set at 100%.

### Cholesterol assay

Cholesterol content in sorted CD45 + /CD11B + cells (*n* = 6–8/group) or in serum of mice (*n* = 6/group) was assessed using the Amplex Red Cholesterol Kit (ThermoFisher, Massachusetts, USA) following manufacturer’s instructions. Data are normalized to protein content with the mean value of the vehicle treated group set at 100%.

### BMDM isolation

Bone derived macrophages were obtained from C57BL/6 J mice (IMSR_JAX:000664**) **(21 month-old mice) (*n* = 3) as previously described [45]. Briefly, femurs and tibia were obtained from mice after euthanasia using isoflurane and cervical dislocation and marrow flushed using RPMI-1640 supplemented with 10% FBS, antibiotics, and glutamine. The fluid containing dissociated marrow cells was passed through a cell strainer. Cells were plated onto coverslips coated with poly-l-lysine in a 12-well plate and allowed to attach overnight in a humidified incubator with 5% CO_2_ at 37 °C. The following day, coverslips were washed 3 times with warm phosphate buffered saline (PBS) to remove non-adherent cells before the media was replaced with RPMI-1640 supplemented with 10% FBS, antibiotics, glutamine, and 10% L929 conditioned media to differentiate the cells. Once fully differentiated (~ day 6), cells were processed for further experiments.

### Immunofluorescence

Fully differentiated macrophages were treated with either vehicle or 1 µM GW3965 for 24 h, then co-incubated with 10 µg/mL DiI-oxLDL (ThermoFisher, Massachusetts, USA) for another 24 h (*n* = 3 experiments in triplicate). Cells were then washed with ice cold PBS and fixed with 4% PFA for 15 min. After washing with PBS, coverslips were co-stained with DAPI and mounted for imaging. Images were captured using Olympus IX80 Inverted Microscope (Olympus Corporation, Massachusetts, USA) equipped with an X-Cite 120Q fluorescent light source (Lumen Dynamics, Ontario, Canada) and a CoolSNAP HQ2 CD camera (Photometrics, Arizona, USA). Image processing and quantification (*n* = 50 cells/group) was performed using CellSens (Olympus Corporation, Waltham, Massachusetts) and Fiji software (SCR_002285).

### Oxygen consumption assay

A Seahorse XFe96 Analyzer (Agilent, California, USA) was used to measure oxygen consumption rate (OCR) [46–48] of macrophages (seeding density 100,000 cells/well) treated with oxLDL and GW3965 as described above. Briefly, 1 h before the assay, culture media was aspirated from the cells in the plate and Seahorse XF assay media supplemented with 20 mM glucose and 2 mM glutamine was applied and equilibrated in a CO_2_ free incubator. Oligomycin (2 µM, final) (inhibits complex V of the electron transport chain (ETC)), carbonyl cyanide-4-(trifluoromethoxy)phenylhydrazone (FCCP, uncoupling agent) (2 µM, final), antimycin A and rotenone (0.75 µM, final) (inhibit complexes I and III, inhibiting ETC activity) were sequentially injected into the system to induce changes in ETC activity. OCR values were normalized to total protein in individual wells (*n* = 15 wells/group).

### Quantification and statistical analysis

All data are represented as Mean ± S.E.M. Analysis and graphing were performed using GraphPad Prism 9.1.2 (SCR_002798; California, USA). For single group comparisons, either a 1- or 2-tailed *t* test was used as appropriate. Multiple comparisons were performed using one-way ANOVA. The number of experiments/replicates and mice for each experiment are described in figure legends.

## Results

### Aged mice developed features of neuropathic pain and small fiber neuropathy.

**Fig. 1:**
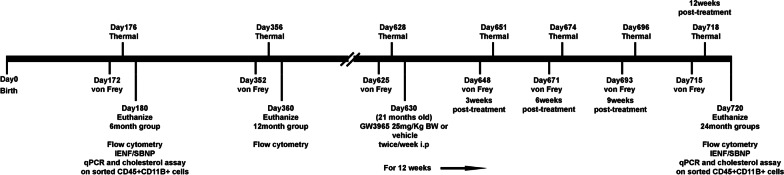
Schematic of experimental design

**Fig. 2:**
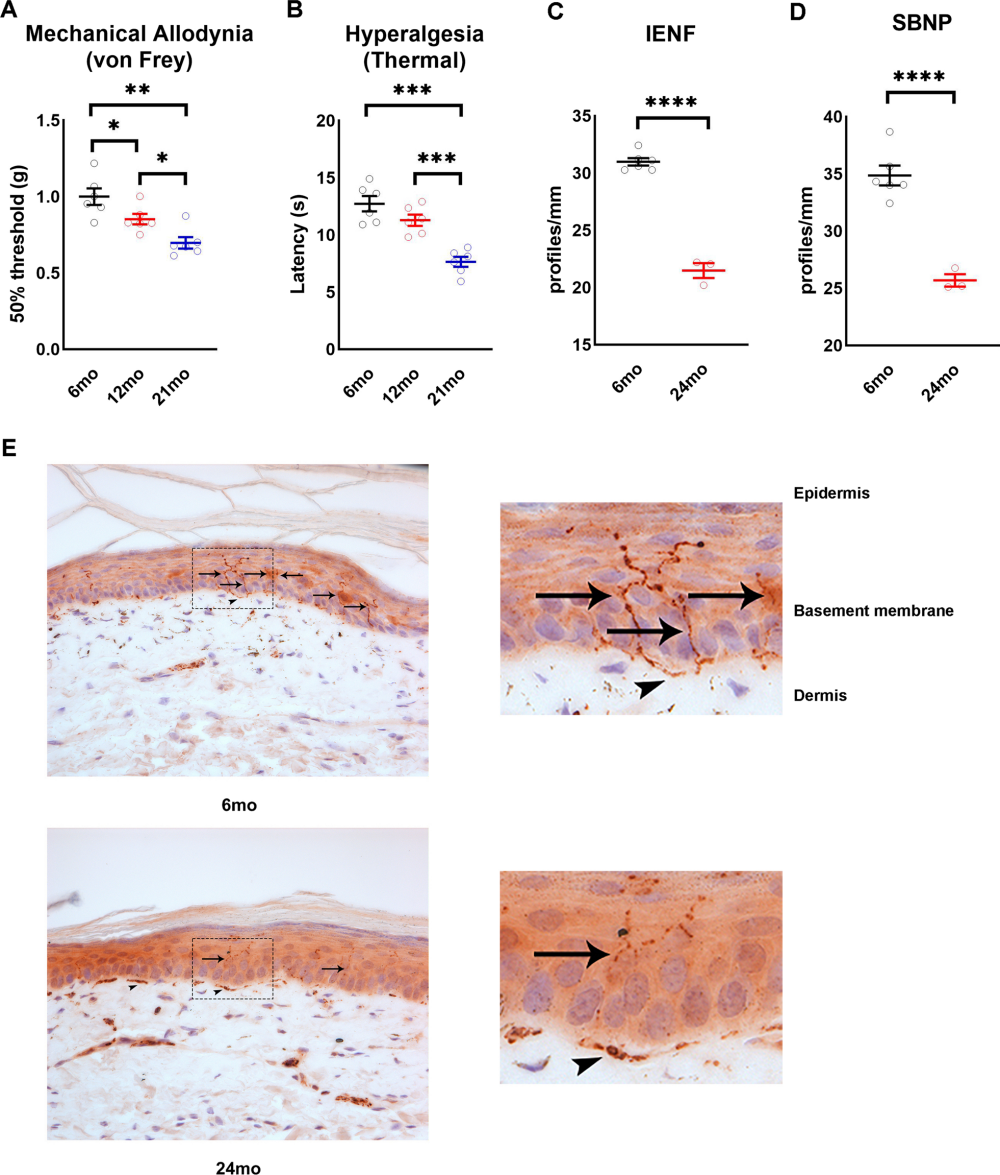
Aging leads to loss of distal nerve and neuropathy. **A** Mechanical allodynia in 6, 12, and 21 month-old mice (*n* = 6/group). **B** Thermal hyperalgesia in 6, 12, and 21 month-old mice (*n* = 6/group). Distal nerve quantification in 6 and 24 month-old mice, IENF **C** and SBNP **D** (*n* = 3–5/group). **E** Representative images of paw plantar skin of 6 and 24 month-old mice used for IENF quantification. PGP9.5 positive nerve fibers stain brown. Black arrows indicate intra-epidermal nerve fibers and arrowheads indicate dermal nerves. All images were obtained at 40 × magnification. All data are Mean ± SEM. *p < 0.05, **p < 0.005, ***p < 0.0005, ****p < 0.00005

We used the von Frey and Hargreaves tests to longitudinally evaluate sensitivity of 6-month, 12-month, and 21-month-old mice (Fig. 1). During the von Frey test, we observed a significant decrease in the 50% response threshold of mice starting at 12 months of age and progressing at 21 months of age (Fig. 2A) (*n* = 6/group), suggesting an increased sensitivity to innocuous stimuli with age. Mice also developed sensitivity to heat, with significant hyperalgesia detected in 21-month-old mice compared to 12-month-old mice (Fig. 2B) (*n* = 6/group). Thus, age significantly affects escape responses to both mechanical and thermal stimuli.

We then quantified sensory nerve density in the epidermis (intraepidermal nerve fibers; IENF and sub-basal nerve plexus; SBNP). We observed a significant decrease in both IENF (Fig. 2C, E) (*n* = 4–6/group) and SBNP (Fig. 2D, E) (*n* = 4–6/group) density in 24 month-old mice compared to 6-month-old mice demonstrating that aging led to distal sensory neuropathy.

### Age-related accumulation of macrophages in the dorsal root ganglia and sciatic nerve

**Fig. 3:**
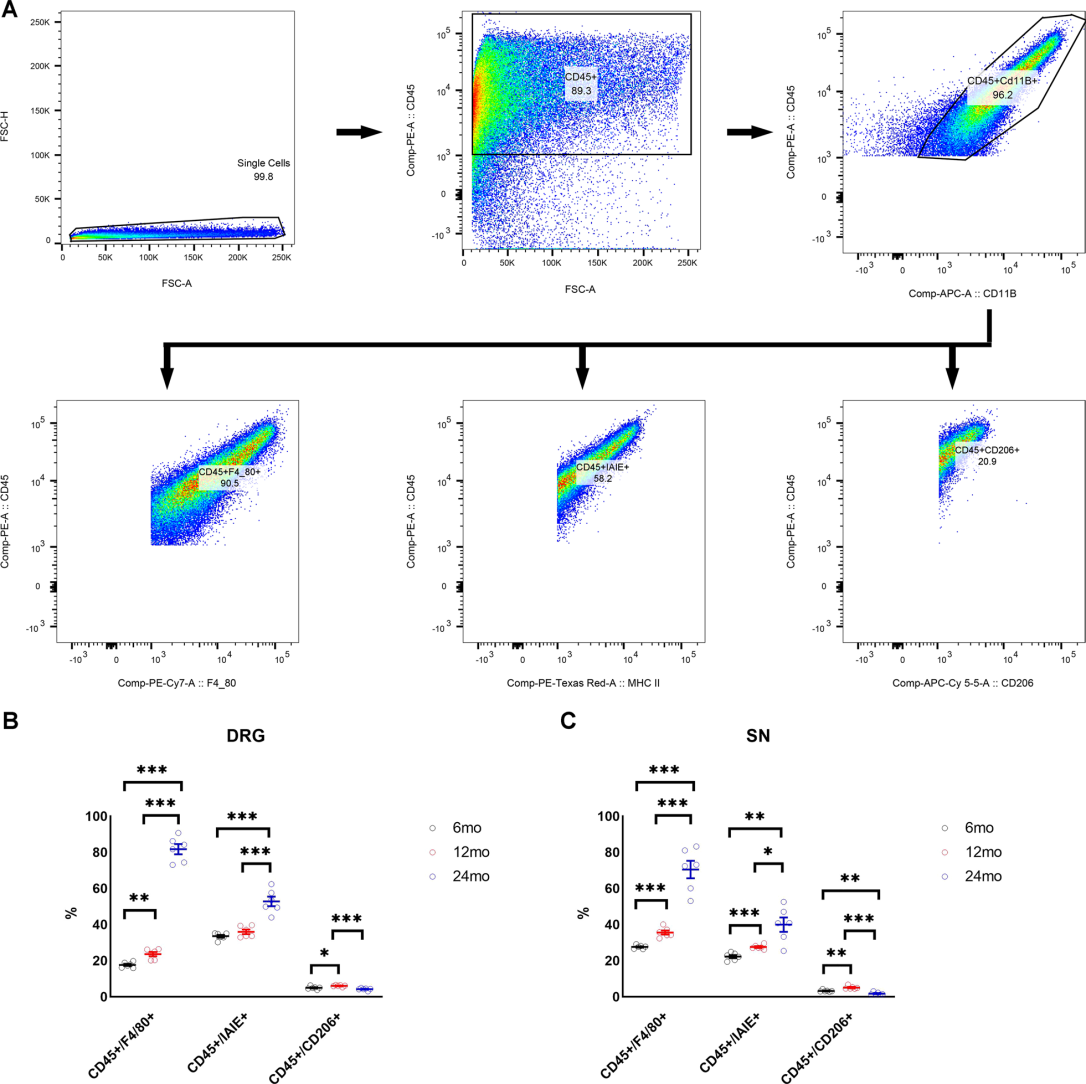
Activated macrophage numbers and their phenotype change with age. **A** Gating strategy used for sorting and quantifying macrophage population. **B** Percentage macrophage population and phenotypes in the DRG of 6, 12, and 24 month-old mice (*n* = 6/group). **C** Percentage macrophage population and phenotypes in the SN of 6, 12, and 24 month-old mice (*n* = 6/group). All data are Mean ± SEM. *p < 0.05, **p < 0.005, ***p < 0.0005

Previous studies in mice have identified macrophages as contributors to assorted peripheral neuropathies [49]. We performed flow cytometry of cells derived from the DRG and SN and used specific cell surface markers to identify macrophage numbers and phenotypes (Fig. 3A). We observed a significant temporal increase in the percentage of cells expressing the pan-macrophage markers CD45 + F4/80 + in both the DRG and SN, with the highest increase occurring between 12 and 24 months of age (Fig. 3B, C) (*n* = 6/group). We also observed a temporal increase in percentage of cells identified as pro-inflammatory macrophages (CD45 + IAIE +) in the DRG and SN (Fig. 3B, C) (*n* = 6/group) and a significant increase in the percentage of potentially anti-inflammatory macrophage M2 (CD45 + CD206 +) (*n* = 6/group) population in both DRG and SN of 12-month-old mice compared to 6-month-old mice (Fig. 3B, C) (*n* = 6/group). In contrast, the proportion of M2 macrophages significantly decreased in the DRG and SN of 24-month-old mice (Fig. 3B, C) (*n* = 6/group).

Comparison of sural nerve biopsies from two individuals (subject 1: 84 years and subject 2: 37 years) demonstrated a considerably lower percentage of M2 macrophages in older individual compared to the younger individual (Additional file 1: Fig. S1A). Similar to mice, we also observed a higher percentage of M1 macrophages in older individual compared to younger individual (Additional file 1: Fig. S1A). These data that will need to be confirmed with larger sample size suggest that phenotype of activated macrophages may change with age.

### LXRs agonist delays progression of age-associated neuropathic pain and neuropathy

**Fig. 4:**
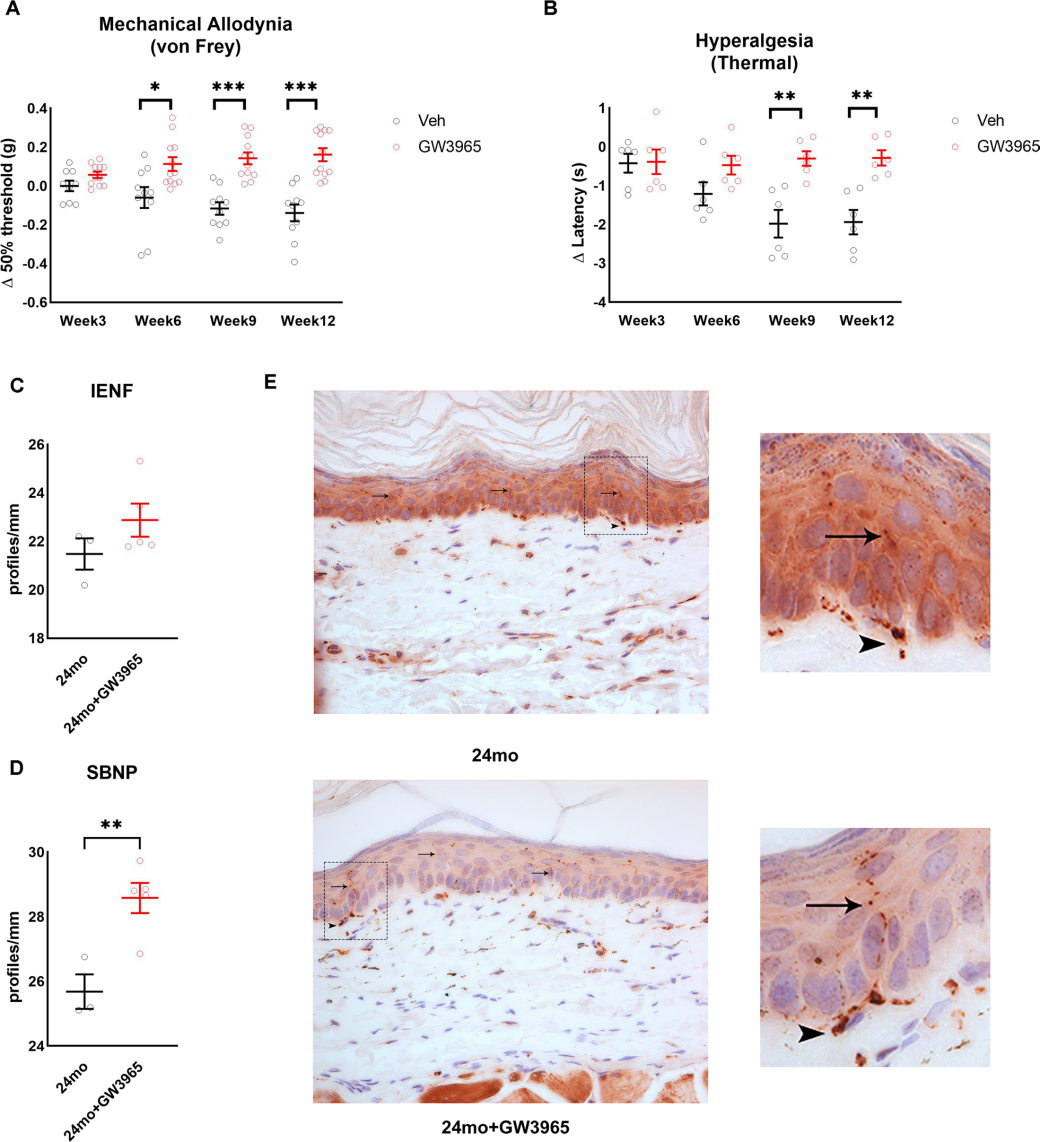
LXRs activation improves indices of age-related neuropathic pain. **A** Change in mechanical allodynia in 24 month-old mice treated with and without LXRs agonist GW3965 for 12 weeks (*n* = 10–12/group). **B** Change in thermal hyperalgesia in 24-month-old mice treated with and without LXRs agonist GW3965 for 12 weeks (n = 6/group). Distal nerve quantification in 24-month-old mice treated with and without LXRs agonist GW3965, IENF **C** and SBNP **D** (*n* = 4–5/group). **E** Representative images of paw plantar skin of 24-month-old mice treated with and without LXRs agonist GW3965 used for IENF quantification. PGP9.5 positive nerve fibers stain brown. Black arrows indicate intra-epidermal nerve fibers and arrowheads indicate dermal nerves. All images were obtained at 40 × magnification. All data are Mean ± SEM. *p < 0.05, **p < 0.005, ***p < 0.0005

We next assessed whether activation of LXRs using its potent and selective agonist GW3965 could change the progression of age-related neuropathic pain and neuropathy. Aged (21 month-old) mice were injected with the LXRs agonist GW3965 (25 mg/kg body weight) or vehicle for 12 weeks [10]. LXRs activation may have systemic effects and as we published before routes and modes of administration would lead to more or less adverse effects. As we and others showed before [10, 34], our treatment paradigm does not lead to hypercholesterolemia and hypertriglyceridemia (Additional file 1: Fig. S1D, E). Compared to vehicle, treatment with GW3965 prevented the further development of mechanical hypersensitivity and heat hyperalgesia over time, with a significant difference observed after 6 weeks of treatment for mechanical sensation (*n* = 10–12/group) and after 9 weeks of treatment for heat sensation (Fig. 4A, B) (*n* = 6/group). We then evaluated the effect of GW3965 on IENF and SBNP density in the paw of these mice. Compared to vehicle treated mice, we observed a trend to increased IENF of 24-month-old mice treated with GW3965 (Fig. 4C, E) (*n* = 3–4/group) and a significant increase in SBNP density in 24-month-old mice treated with GW3965 (Fig. 4D, E) (*n* = 3–4/group). These data suggest that LXRs activation either attenuated distal degeneration or stimulated nerve regeneration and demonstrate that in vivo activation of LXRs using GW3965 can improve indices of age-related neuropathic pain and sensory neuropathy.

### Effect of LXRs agonist treatment on macrophage in the DRG and SN

**Fig. 5:**
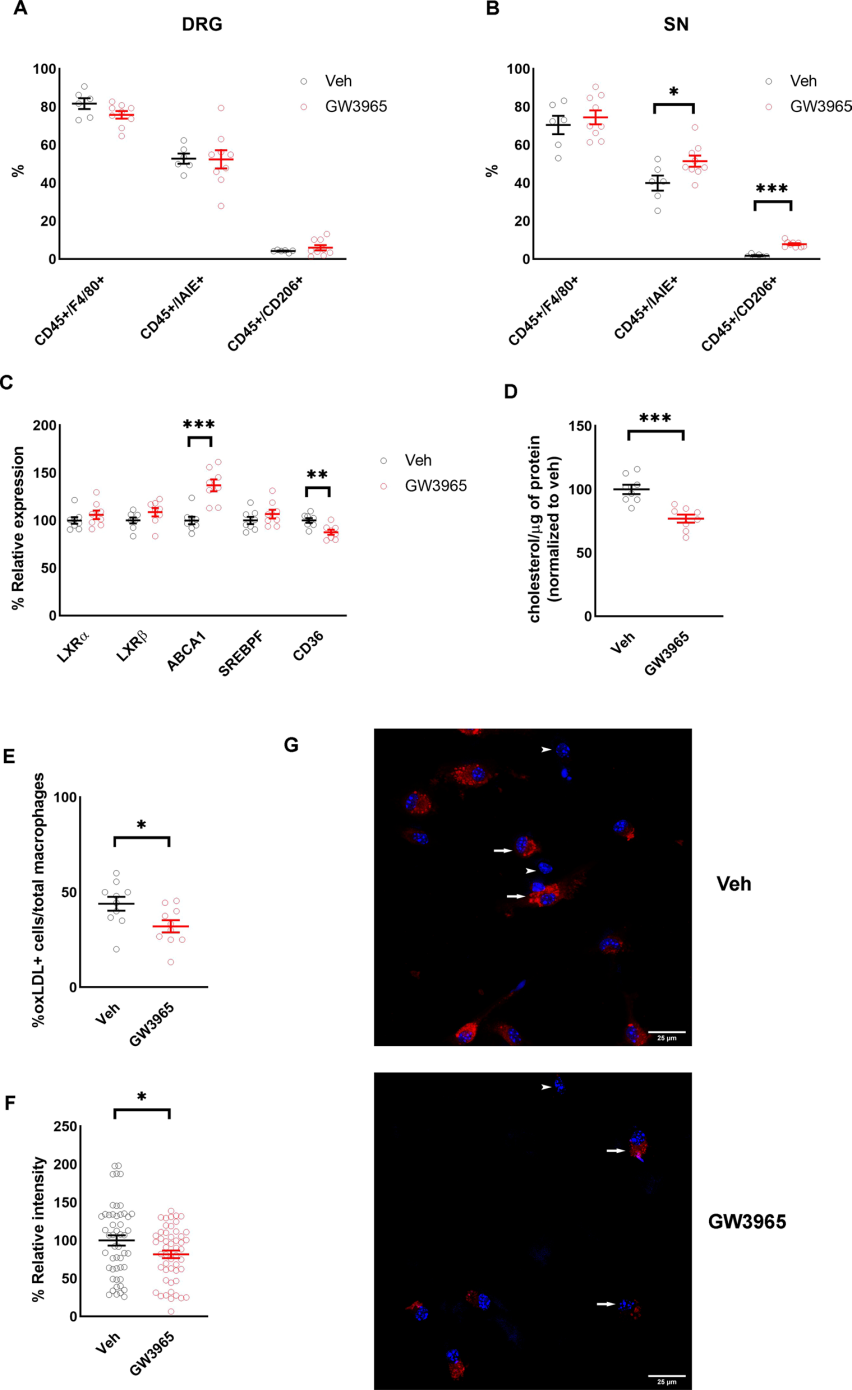
Activation of LXRs increases lipid efflux. **A** Percentage macrophage population and phenotypes in the DRG of 24-month-old mice treated with and without LXRs agonist GW3965 (*n* = 6–9/group). **B** Percentage macrophage population and phenotypes in the SN of 24-month-old mice treated with and without LXRs agonist GW3965 (*n* = 6–9/group). **C** mRNA expression of LXRs target genes in the sorted CD45 + CD11B + cells from the SN of 24-month-old mice treated with and without LXRs agonist GW3965 (*n* = 8/group). **D** Cholesterol content in the sorted CD45 + CD11B + cells from the SN of 24-month-old mice treated with and without LXRs agonist GW3965 (*n* = 8/group). **E** Percentage oxLDL positive cells from macrophages treated with and without LXRs agonist GW3965 (*n* = 3 experiments in triplicate). **F** oxLDL relative intensity in oxLDL positive cells from macrophages treated with and without LXRs agonist GW3965 (*n* = 50 cells/group). **G** Representative images of oxLDL positive cells from macrophages treated with and without LXRs agonist GW3965. Arrows represent macrophages with oxLDL staining (oxLDL-red, nucleus-DAPI/blue) and arrowheads represent macrophages without oxLDL staining. All data are Mean ± SEM. *p < 0.05, **p < 0.005, ***p < 0.0005

To assess whether LXR activation modifies PNS immune cells we quantified some immune cells in the DRG and SN using flow cytometry. Although there was not a significant change in proportions of any macrophage phenotype in the DRG (Fig. 5A) (*n* = 8/group), we did observe a significant increase in percentage of both pro-inflammatory (CD45 + IAIE +) M1 macrophages and anti-inflammatory (CD45 + CD206 +) M2 macrophages in the SN of mice treated with GW3965 (Fig. 5B) (*n* = 8/group).

Transcript levels of multiple lipid efflux mediators showed an age-related decrease (Additional file 1: Fig. S1B) (*n* = 8/group). LXRs play an important role in regulating macrophage cholesterol efflux in other tissues [32, 33] but their role in regulation of cholesterol in macrophages of peripheral nerves had not to our knowledge been evaluated. We sorted CD45 + CD11B + cells from the DRG and SN and studied Abca1, Srebpf (Sterol regulatory element-binding transcription factor 1) and Cd36 (cluster of differentiation 36, scavenger receptor) transcript levels, these genes are involved in lipid de novo production and lipid efflux. We found a significant increase in expression of Abca1 and a significant decrease in expression of CD36 in cells harvested from the SN of LXR agonist-treated mice (Fig. 5C) (*n* = 8/group). We also found significantly decreased cholesterol content in sorted cells from mice treated with GW3965 compared to vehicle treated mice (Fig. 5D) (*n* = 8/group). Differentiated macrophages derived from bone marrow of old mice were culture in presence of oxidized low-density lipoproteins (oxLDL) to obtaining many lipid-loaded cells [50, 51], and then treated with GW3965. Consistent with data on SN and literature [52, 53], we found a significantly lower percentage of cells labeled with oxLDL in GW3965 treated group (Fig. 5E, G) (*n* = 3 experiments in triplicate). Within the cells that were positive for oxLDL, GW3965 treated cells had significantly lower content of oxLDL compared to vehicle treated cells (Fig. 5F, G) (*n* = 50 cells/group).

**Fig. 6:**
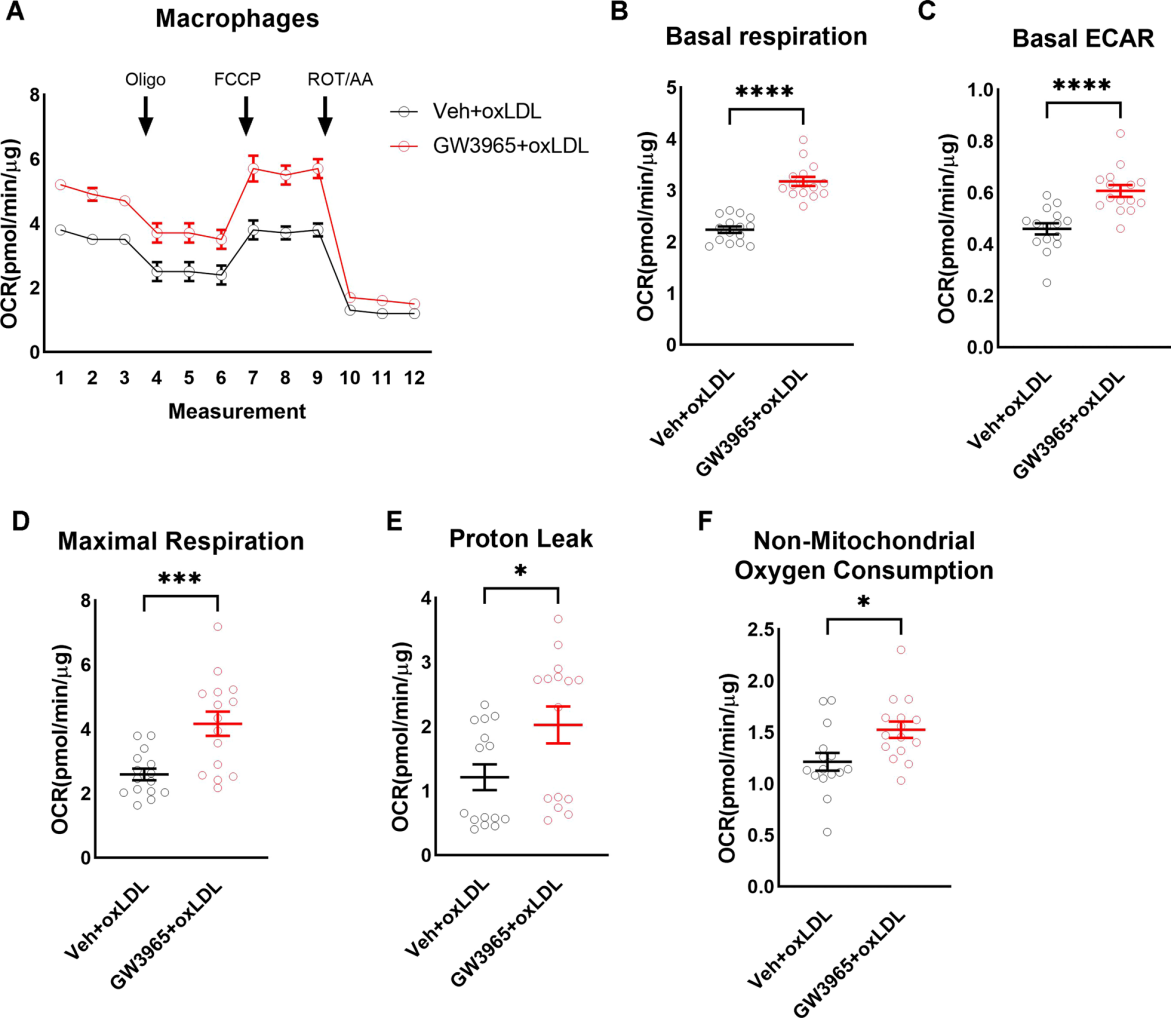
Activation of LXRs is associated with change in macrophage polarization. During oxygen consumption assay in the presence of oxLDL and with and without LXRs agonist GW3965 (**A**), LXRs activation significantly increased basal respiration (**B**), basal ECAR (**C**), maximal respiration (**D**), proton leak (**E**), and non-mitochondrial oxygen consumption **F** (*n* = 15 wells/group). All data are Mean ± SEM. *p < 0.05, ***p < 0.0005, ****p < 0.00005

To test if LXRs activation and potentially lipid content affected macrophage metabolism, we measured oxygen consumption rate (OCR) in differentiated macrophages derived from bone marrow of old mice with or without concurrent GW3965 (Fig. 6A) (*n* = 15 wells/group). Compared to vehicle, LXRs agonist treatment significantly increased basal respiration (Fig. 6B) (*n* = 15 wells/group) and basal glycolytic activity (basal ECAR, Fig. 6C) (*n* = 15 wells/group). LXRs activation also significantly increased maximal respiration (Fig. 6D) (*n* = 15 wells/group), proton leak (Fig. 6E) (*n* = 15 wells/group), and non-mitochondrial oxygen consumption (Fig. 6F) (*n* = 15 wells/group), without having a significant effect on spare respiratory capacity, coupling efficiency, or ATP-linked oxygen consumption (not shown). These data suggest that LXRs activation changes the metabolism of macrophages exhibiting energetic phenotype.

## Discussion

Aging is a major risk factor for damage to the structure and function of the PNS [54]. We observed a significant decrease in the mechanical threshold and latency for thermal hyperalgesia of mice with age, suggesting an increased sensitivity to innocuous stimuli with age. Here, we confirm previous studies showing that aged mice (24 months) exhibit sensory dysfunction, loss of distal sensory fibers and elevated endoneurial macrophages [6, 55, 56]. Endoneurial macrophages, both resident and invading, are widely studied in the context of their capacity to phagocytose debris following physical nerve injury, a process that paves the way for nerve regeneration, while dysregulation of the immune system can prompt macrophages to inappropriately attack Schwann cells and myelin in autoimmune and inflammatory neuropathies. [21–24]. Inflammatory response develops rapidly after peripheral nerve injury, which contributes to both neuropathic pain and nerve regeneration [14]. Therefore, it is crucial to study immune cells in pathophysiology or after nerve injury to identify new innovative targets for treating neuropathic pain. Both circulating and resident macrophage populations may contribute to the pathogenesis of PNS injuries. Resident macrophages are the early responders upon peripheral nerve injury, digesting myelin as soon as 2 days after sciatic nerve crush [57]. Over time, the ratio of number of infiltrating macrophages outweighs resident macrophages following nerve injury, but little is known whether they have distinct functions in the PNS and in aging [58]. Resident macrophages in the PNS possess a microglia-like phenotype, which includes the removal of debris, inflammation, and remyelination during injury [59]. Intriguingly, a contribution of macrophages to the pathogenesis of age-related neuropathy has recently been suggested by a report that silencing of foamy macrophages in the PNS improved the nerve structure and function of aged mice [6]. Despite evidence for a role of macrophages loaded with lipid in the development of age-related neuropathy, the mechanism is not known. The presence of phagocyting macrophages, while necessary for a healthy nerve structure and function, may also induce nerve damage via dysfunction in lipid efflux and clearance by phagocytic immune cells.

We have previously reported that activation of LXR in the sensory neurons of the DRG is required for amelioration of obesity-induced allodynia, and that selective deletion of LXRs from sensory neurons enhances the neuropathic pain phenotype [10]. LXRs agonist, GW3965, has been shown to promote the recovery of damaged nerves in many acute models of nerve injury. Indeed, administration of GW3965 following sciatic nerve crush prevents the development of mechanical allodynia and is concomitant with an increase in M2-phenotype of resident macrophages, thus contributing to the resolution of inflammation and promotion of tissue repair [60]. In a mouse model of demyelination using lysolecithin injection, GW3965 promotes myelin debris clearance through the upregulation of LXR-regulated cholesterol transport genes in macrophages, including ApoE, Abca1, and Abcg1 [61]. We found that the LXR selective agonist GW3965 either reversed or delayed the progression of multiple functional and structural indices of neuropathy observed in aged mice. Treating 24-month-old mice with GW3965 prevented first the development of mechanical hypersensitivity and then thermal hyperalgesia over time. This improvement in pain indices was also accompanied with an increase in SBNP density suggesting LXRs activation either attenuated distal degeneration or stimulated nerve regeneration. This is consistent with our prior studies that showed LXR agonist treatment prevents progression of obesity-induced allodynia by altering peripheral sensory neuron function and their interaction with associated cells [10, 62]. Peripheral macrophages have been implicated in the development of mechanical allodynia [63] and infiltration by immune cells happen differently in myelinated or non-myelinated fibers that may explain temporal differences in sensory behavior. In addition, a recent report showed that transcriptional changes in DRG caused by nerve injury correlated with temporal changes in nocifensive behavior—changes observed in nociceptor-related transcripts correlated with the early development of thermal allodynia, while changes observed in immune-related genes correlated with the later development of tactile allodynia [63]. These findings may be pertinent to the differences we observed in normalization of mechanical and thermal sensitivities in aging. We also observed that LXR agonist increases the fiber density in the skin of aged mice. Loss of IENF is usually associated with neuropathic pain, many reports suggest various mechanisms but there is still a lack of consensus that explains why less fibers lead to pain [64, 65]. Many reports converge toward the conclusion that while some neurons are retracting, neurons become hyperexcitable for various reasons, leading to pain. Previous studies have shown IENF loss in patients with diabetes, but was also observed in patients with diabetes but without pain [65]. This loss of IENF does not explain pain. It is possible, however, that patients with pronounced loss would develop these pain symptoms later. Others have reported a specific relationship between loss of IENF and changes in thermal perception, but found that pain and aberrant thermal perception resolved before IENFs reinnervated the skin [66]. There are likely distinct mechanisms regulating the loss of IENF and progression of neuropathic pain and crosstalk between these pathways may occur in some cases. While more investigation would be necessary to establish causality, our findings suggest that activation of LXRs influence sensory perception and nerve degeneration/regeneration of the aged mice via modifying macrophages of the SN. Our exploratory study using two human sural nerves suggests an age-related difference in macrophage polarization and encourages future additional studies using larger numbers.

LXRs are oxidized cholesterol derivative ligand-activated transcription factors and consist of two isoforms LXRα and LXRβ [10, 30, 31, 62]. LXRs play an important role in cholesterol metabolism [32, 33]. Several studies have reported that activation of LXRs inhibit the development of atherosclerosis, a property attributed to LXR-mediated ABCA1 expression and cholesterol efflux in macrophages [52, 53]. LXRs also play an important role in the regulation of cytokine production and the anti-inflammatory response [32, 33]. In this study, we provide evidence supporting the potential role of LXR activation in the regulation of PNS macrophage cholesterol content. A number of anti-inflammatory mechanisms have been proposed to explain the actions of LXRs in other tissues including direct repression of pro-inflammatory gene promoters, cholesterol efflux, changes in plasma membrane signaling systems via modulation of membrane lipid composition, and increased synthesis of fatty acids with anti-inflammatory activity [32, 67, 68]. The engulfment of myelin and other cellular debris in injured nerve by macrophages is associated with acute changes in their cellular lipid levels [35], including intracellular-free cholesterol. Activation of LXRs during this process promotes the efflux of free cholesterol by activation of ABCA1 [52, 53].

Our data demonstrate that the amount of cholesterol within macrophages sorted from the SN of aged mice is decreased after activation of LXR. An emerging body of evidence indicates that cellular metabolism determines macrophage function as either pro- or anti-inflammatory [69]. However, the role of lipid content in PNS macrophage polarization is unknown. We failed to obtain enough macrophages from SN of mice so to assess macrophages' bioenergetic profiles, so we used aged bone-marrow-derived macrophages cultured in oxLDL to maximize the lipid-loading of the cells. After treatment with the LXRs activator GW3965, we find that these macrophages increase both OCR and ECAR. This metabolic flux results imply that both glycolysis (ECAR) and mitochondrial respiration (OCR) are upregulated in macrophages treated with GW3965, potentially mimicking the energetic M2 phenotype [69]. Altogether, our data indicate that activation of LXRs in the PNS could induce phenotypic alteration of immune cells—by restoring or activating the lipid efflux in peripheral macrophages—resulting in a sensory and neuropathy improvement in aged mice.

## Conclusions

We demonstrated that neuropathic alterations in aging mice were accompanied by changes in macrophage profiles. Even though lacking power, we saw similar data in human sural nerve. LXR agonist, GW3965, can delay age-related alterations to mechanical hypersensitivity and hyperalgesia, and increase sensory nerve fibers of the skin potentially resulting from an LXR-dependent regulation of macrophage cholesterol/lipid metabolism. However, future studies are required to dig deeper into comprehensive molecular mechanisms linking PNS/lipid/macrophage and to investigate whether LXRs activation attenuated distal degeneration or stimulated nerve regeneration in aging mice. Metabolic syndrome and neuropathy are closely associated and has been shown to be one of the driving factor for nerve injury in aged population by increasing fatty deposition in nerves, mitochondrial dysfunction, and oxidative stress [70]. Activation of these pathways also leads to metabolic inflammation [70]. Further studies in lipid metabolism of immune cells/macrophages of aging mice may advance our understanding of the pathophysiology of age-related neuropathic pain and help us in increasing the quality of life in the elderly.

## Supplementary Information

**Additional file 1: Fig. S1.** **A** Percentage of M1 (CD45 + /HLA-DR +) and M2 (CD45 + /CD206 +) macrophages in human sural nerve biopsies from 37 and 84yrs old female patients. **B** mRNA expression of LXRs target genes in the sorted CD45 + CD11B + cells from the SN of 6-month-old and 24-month-old mice (*n* = 8/group). **C** Cholesterol content in the sorted CD45 + CD11B + cells from the SN of 6, 12, and 24 month-old mice (*n* = 8/group). Cholesterol **D** and triglycerides **E** levels of 24-month-old mice treated with and without LXRs agonist GW3965 for 12 weeks (*n* = 6/group). All data are Mean ± SEM. *p < 0.05, **p < 0.005, ***p < 0.0005, ****p < 0.00005.

## Data Availability

All data generated or analyzed during this study are included in this published article [and its Additional file 1: Fig. S1].

## Abbreviations

ABCA1: ATP binding cassette transporter A1
ATP: Adenosine triphosphate
CD11B: Cluster of differentiation 11b, leukocyte marker
CD206: Cluster of differentiation 206, M2 macrophage marker
CD36: Cluster of differentiation 36, scavenger receptor
CD45: Cluster of differentiation 45, lymphocyte common antigen
DAPI: Diamidino-2-phenylindole
Dil-oxLDL: DiI dye oxidized low-density lipoproteins
DRG: Dorsal root ganglia
ECAR: Extracellular acidification rate
ETC: Electron transport chain
F4/80: Macrophage marker
FBS: Fetal bovine serum
FCCP: Carbonyl cyanide-4-(trifluoromethoxy)phenylhydrazone
I-A/I-E: MHC class II marker
IENF: Intraepidermal nerve fibers
LXR: Liver X receptor
OCR: Oxygen consumption rate
oxLDL: Oxidized low-density lipoproteins
PBS: Phosphate buffered saline
PNS: Peripheral nervous system
RPMI-1640: Roswell park memorial institute medium-1640
SBNP: Sub-basal nerve plexus
SN: Sciatic nerve
SREBPF: Sterol regulatory element-binding transcription factor 1
