# Supplementary figures and images for: LXR agonist improves peripheral neuropathy and modifies PNS immune cells in aged mice
Source: J Neuroinflammation. 2022 Feb 26;19:57. doi: 10.1186/s12974-022-02423-z (PMC8882298; doi:10.1186/s12974-022-02423-z)

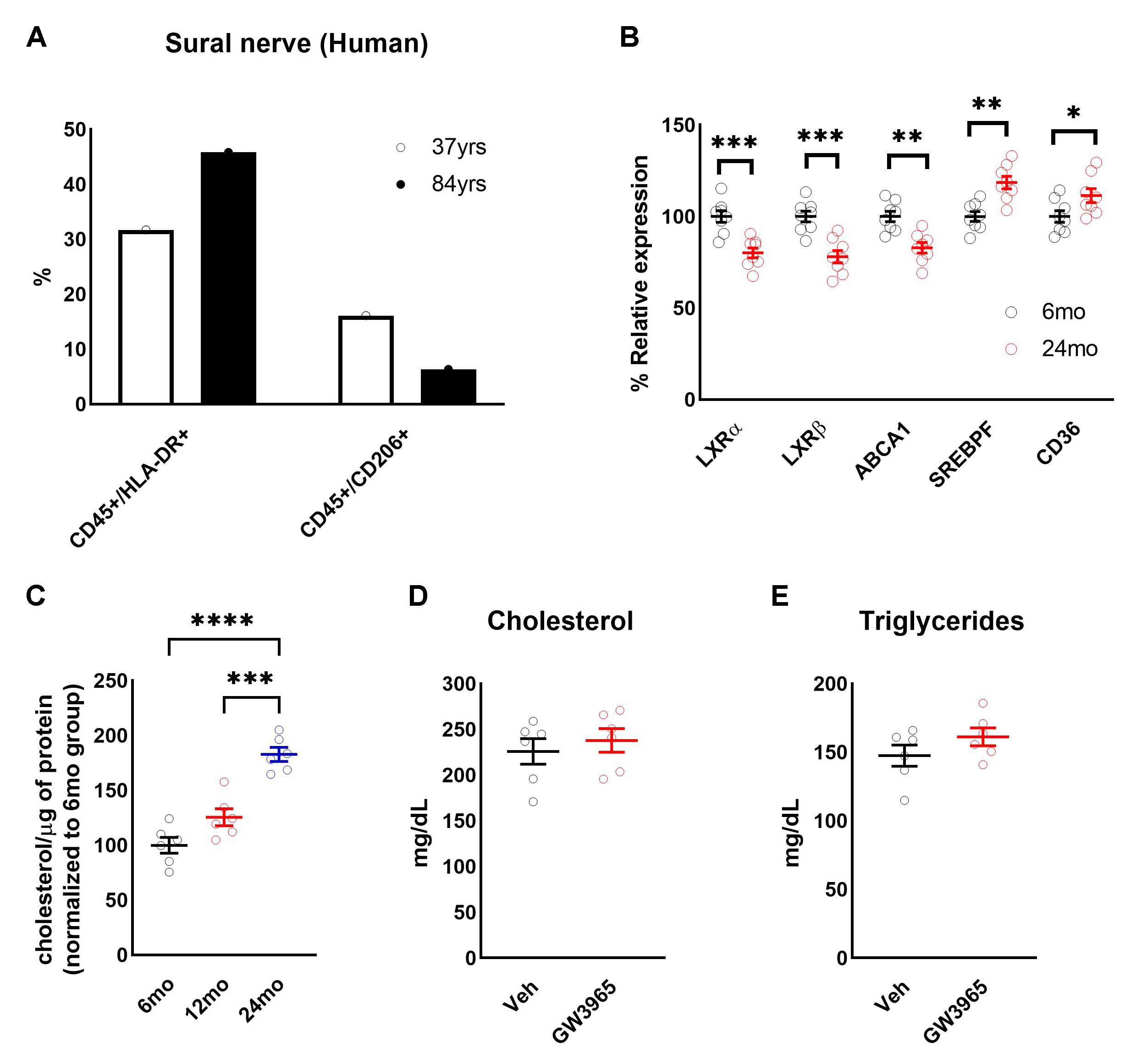

Supplement: Supplementary file 1 — Additional file 1: Fig. S1. A Percentage of M1 (CD45 + /HLA-DR +) and M2 (CD45 + /CD206 +) macrophages in human sural nerve biopsies from 37 and 84yrs old female patients. B mRNA expression of LXRs target genes in the sorted CD45 + CD11B + cells from the SN of 6-month-old and 24-month-old mice (n = 8/group). C Cholesterol content in the sorted CD45 + CD11B + cells from the SN of 6, 12, and 24 month-old mice (n = 8/group). Cholesterol D and triglycerides E levels of 24-month-old mice treated with and without LXRs agonist GW3965 for 12 weeks (n = 6/group). All data are Mean ± SEM. *p < 0.05, **p < 0.005, ***p < 0.0005, ****p < 0.00005. [file 12974_2022_2423_MOESM1_ESM.jpeg]
